# Regulatory mechanisms of exercise-induced physiological cardiac hypertrophy: progress and prospects

**DOI:** 10.3389/fcvm.2025.1657950

**Published:** 2025-09-25

**Authors:** Peng Cheng, Xi Zhang, Yi Si, Qiushi Yin, Lin Chen, Qin Ru, Chong Chu, Hongyue Xiang, Ling Liao, Hang Ran, Zaihong Zhang, Yuxiang Wu

**Affiliations:** ^1^ Institute of Intelligent Sport and Proactive Health, Department of Health and Physical Education, Jianghan University, Wuhan, China; ^2^ College of Sports Medicine, Wuhan Sports University, Wuhan, China; ^3^ Department of Cardiovascular Surgery, Union Hospital, Tongji Medical College, Huazhong University of Science and Technology, Wuhan, China

**Keywords:** exercise, physiological cardiac hypertrophy, regulatory mechanisms, cardiovascular health, research progress

## Abstract

**Introduction:**

Exercise-induced physiological cardiac hypertrophy (PCH) plays a significant role in cardiovascular health. Although substantial progress has been made in recent years, the precise regulatory mechanisms underlying this adaptive remodeling remain incompletely elucidated and warrant further investigation.

**Methods:**

The literature retrieval and selection process in this study adhered to the PRISMA guidelines. Databases such as Web of Science, PubMed, Embase, and the Cochrane Library were searched, with the retrieval period covering from the establishment of the respective databases up to August 2025. Keywords used in the search included “exercise”, “physiological cardiac hypertrophy”, “assessment methods”, “regulatory mechanisms”, and “cardiovascular health”. Inclusion criteria were: (1) studies exploring the regulatory mechanisms or health effects of exercise on physiological cardiac hypertrophy; (2) studies involving healthy adults (≥18 years) or wild-type animal models (e.g., C57BL/6 mice); (3) studies employing quantitative imaging, laboratory, or electrophysiological methods to assess physiological cardiac hypertrophy. Exclusion criteria included studies focused solely on pathological cardiac hypertrophy, experimental studies lacking a control group, and studies assessed as having a high risk of bias. Literature selection was independently performed by two researchers, and the final eligible studies were systematically summarized.

**Results:**

This review first outlines the definitions, characteristics, and clinical evaluation methods of PCH. It then examines the impact of different exercise modalities on cardiac remodeling and summarizes the underlying regulatory mechanisms, including transcriptional pathways (e.g., IGF-1/PI3K/Akt, NRG1/ErbB signaling), post-transcriptional processes (e.g., RNA m6A methylation and noncoding RNA regulation), and metabolic adaptations (e.g., fatty acid oxidation and glucose utilization).The beneficial effects of exercise-induced physiological cardiac hypertrophy on cardiovascular health are also thoroughly analyzed.

**Discussion:**

Despite its benefits, several challenges remain. Distinguishing PCH from pathological cardiac hypertrophy (PMH) remains difficult, given the limitations of current imaging techniques and biomarkers. Moreover, excessive exercise may precipitate cardiac decompensation, arrhythmias, or dysfunction. Future research should therefore prioritize the development of personalized exercise prescriptions, refinement of diagnostic technologies, and elucidation of the molecular mechanisms driving cardiac decompensation. Such efforts will not only deepen the scientific understanding of exercise-related cardiac remodeling but also provide practical guidance for athlete training and cardiovascular disease prevention.

## 1 Introduction

Cardiovascular health is a fundamental determinant of overall human health and quality of life, serving as the cornerstone of normal cardiac and vascular function. It encompasses multiple dimensions, including myocardial contractile capacity, vascular regulation, and circulatory efficiency. Maintaining optimal cardiovascular health not only ensures adequate perfusion and organ function but also substantially lowers the risk of cardiovascular disease (CVD), which remains the leading cause of morbidity and mortality worldwide (1). Moderate exercise can promote cardiovascular health, enhance skeletal muscle function, and slow aging, with its effects depending on the body's adaptation to regular physical activity (2, 3). Lately, exercise has surfaced as a non-pharmacological method to improve heart health, with adaptive cardiac remodeling has become a key research focus in cardiovascular health (4).

Myocardial hypertrophy (MH) is the myocardium's compensatory response to overload from one or more stimuli, including PMH and PCH. PMH is most commonly induced by conditions such as hypertension or aortic valve stenosis. It is characterized by disorganized cardiomyocyte proliferation, interstitial fibrosis, and impaired contractile function. This maladaptive process is typically accompanied by progressive deterioration of cardiac performance, ultimately predisposing patients to adverse events including arrhythmia and heart failure. Imaging-based diagnostic criteria often reveal abnormal thickening of the left ventricular wall and ventricular dilatation (5). PCH represents an adaptive response of the myocardium to stimuli such as growth, exercise, or pregnancy. Rather than being associated with dysfunction, PCH reflects optimized cardiac performance and structural remodeling that enhances cardiovascular health and, in athletes, improves exercise capacity (6). Diagnostic evaluation of PCH generally relies on multimodal approaches, including advanced imaging, laboratory testing, and electrophysiological assessment. Characteristic findings include left ventricular wall thickening and chamber enlargement without evidence of fibrosis, diastolic dysfunction, or adverse remodeling (7).

As sports medicine research progresses, notable strides have been achieved in comprehending exercise-induced PCH. Yet, its regulatory mechanisms remain incompletely understood. Through a systematic analysis of how different exercise modes impact cardiac structural and functional remodeling, this article delivers an extensive summary of the molecular regulatory mechanisms underlying exercise-induced PCH. It also delves into the benefits of exercise-induced PCH for cardiovascular health and the current challenges in this area. The goal is to offer theoretical guidance for athlete training and CVD prevention and treatment.

## 2 Concepts and characteristics of PCH

### 2.1 Overview of PCH

PCH is primarily characterized by moderate ventricular wall thickening, enlarged cardiac chamber volumes, and enhanced pumping function. It typically lacks cardiomyocyte apoptosis or cardiac dysfunction, thus exhibiting cardioprotective effects (6–8). Histologically, PCH is marked by enlarged cardiomyocyte size, orderly arrangement of myocardial fibers, increased sarcomere numbers, and possibly moderate extracellular matrix (ECM) remodeling (9). This physiological adaptation was first termed “athlete's heart” in the late 19th century by Swedish researcher Henschen through clinical observations of cross-country skiers. Subsequent studies have identified three key features: exercise-induced bradycardia, exercise induced PCH, and compensatory enhancement of cardiac function (10). Extensive research has demonstrated that PCH fundamentally differs from PMH, with their distinguishing characteristics summarized in Table 1.

**Table 1:** Comparative characteristics of PCH and PMH.

Classification dimension	Feature type	PCH	PMH	References
Etiology	Triggering Mechanism	Growth and development, exercise, pregnancy, etc.	Hypertension, aortic valve stenosis, etc.	(6, 11–14)
Morphology	Hypertrophy Pattern	Uniform symmetry	Asymmetry	(15–18)
	Myocyte Arrangement	Ordered arrangement	Disordered arrangement	(19–22)
	Interstitial Fibrosis	None	Significant	(23–25)
	Mass and Volume Changes	Increased	Increased	(6, 26, 27)
Cell Dynamics	Calcium Ion Regulation	enhanced	dysfunction	(6, 14)
	Cell Apoptosis	Inhibited	Activated	(6, 13, 23)
	Contractile Function	Enhanced	Decreased	(6, 14, 26)
Metabolism	FAO	Increased	Decreased	(23, 26, 28)
	Glucose Oxidation	Increased	Increased	(23, 26, 28–30)
Gene Expression	Embryonic gene reprogramming	Normal	Upregulation	(29, 31, 32)

### 2.2 Clinical evaluation of PCH

#### 2.2.1 Radiological evaluation

Imaging assessment of PCH can be performed using advanced modalities such as three-dimensional echocardiography (3DE), tissue Doppler imaging (TDI), and cardiac magnetic resonance imaging (CMR), which allow for accurate evaluation of cardiac structure, function, and remodeling. 3DE offers a robust approach reconstructing cardiac anatomy by capturing volumetric data in real time. One of its key advantages is the ability to accurately measure left ventricular ejection fraction (LVEF), a critical parameter in the assessment of PCH (33). In healthy individuals, LVEF typically falls within the range of 55%–70%. During PCH, the left ventricular end-diastolic volume (LVEDV) increases significantly, often accompanied by a compensatory rise in stroke volume (SV). Despite the volumetric expansion, LVEF remains within the normal range or may even exhibit a mild increase, indicating preserved or augmented systolic performance (13, 34, 35). This coordinated volume expansion is associated with the sustained activation of the Insulin-like Growth Factor 1 (IGF-1)/Phosphatidylinositol 3-Kinase (PI3K)/Protein Kinase B (Akt) signaling pathway, which in turn leads to uniform myocardial thickening rather than localized hypertrophy (36). In addition to LVEF, the left ventricular mass (LVM) and the left ventricular mass index (LVMI) also serve as crucial indicators for evaluating PCH. Reference values suggest normal LVM ranges from 73 to 115 g in males and 62–95 g in females, with normal LVMI thresholds of ≤115 g/m^2^ and ≤95 g/m^2^, respectively. In individuals with PCH, both LVM and LVMI show mild increases that remain below the diagnostic thresholds for PMH, though considerable inter-individual variability may be observed. The molecular basis involves increased protein synthesis regulated by the mammalian target of rapamycin complex 1 (mTORC1) and limited myocardial cell proliferation mediated by the Hippo-YAP signaling pathway (35, 37, 38). Despite the advantages of 3DE in providing detailed insights into cardiac structure and function, the technique is subject to several limitations. These include susceptibility to patient-specific factors, greater computational and storage requirements, and relatively lower temporal and spatial resolution compared to other imaging methods.

TDI is an echocardiographic technique that quantifies myocardial motion by detecting Doppler frequency shifts generated by tissue movement, thereby enabling the construction of energy, velocity, and acceleration maps. This method allows for accurate assessment of both systolic and diastolic cardiac function (39). Key functional parameters derived from TDI include the systolic s' wave and the diastolic E/e' ratio. The s' wave reflects the peak velocity of myocardial contraction during systole and exhibits a strong correlation with the LVEF. In healthy individuals, the lateral mitral annular s' wave typically ranges between 7 and 10 cm/s, although this lower boundary may be as low as 5.4 cm/s depending on factors such as age, sex, and equipment calibration (40–42). In case of exercise-induced PCH, the s' wave generally remains within normal levels or shows a modest increase (43). The E/e’ ratio serves as a surrogate marker for estimating the left ventricular filling pressure (LVFP). An E/e’ ratio below 8 is indicative of normal LVFP, whereas values between 8 and 15 fall into an intermediate zone that necessitates supplementary clinical parameters—such as left atrial volume (LAV)—for accurate interpretation. In the setting of PCH, this ratio may exhibit a slight elevation, reflecting adaptive modifications in diastolic function (44–46). This phenomenon reflects adaptive remodeling through the Calcineurin (CaN)/Nuclear Factor of Activated T-cells (NFAT) signaling pathway, which facilitates the rapid Ca^2+^ reuptake process. When the E/e’ ratio is in the “gray zone” accompanied by a decrease in the s' wave, it indicates dysfunction of the PI3K/Akt-m^6^A methylation axis, suggesting potential pathological risks (17). TDI offers several advantages, including high temporal resolution, real-time quantitative assessment, and broad clinical applicability. Nonetheless, its limitations—such as angle dependency and the need for high frame rates—must be taken into account when interpreting results.

CMR is based on the principle of nuclear magnetic resonance, whereby hydrogen protons within myocardial tissue resonate under a strong magnetic field and radiofrequency pulses. The resulting signals are reconstructed into high-resolution, multi-plane, and multi-parameter images, enabling detailed assessment of cardiac morphology and function. Owing to its superior spatial resolution and tissue characterization, CMR is regarded as the gold standard for evaluating myocardial structure and function, and it plays a pivotal role in the clinical assessment of PCH (47). CMR allows precise quantification of parameters such as myocardial thickness, ventricular volumes, and ejection fraction, thereby providing robust diagnostic support. For example, measurements of left ventricular wall thickness (LVWT) are commonly used to evaluate the extent of myocardial hypertrophy (MH). In healthy adults, LVWT is typically 9–10 mm and interventricular septal thickness (LVST) 10–12 mm. In PCH, LVWT and LVST may increase to approximately 13–16 mm, usually accompanied by enhanced diastolic function (E/e’ ≤8), preserved myocardial tissue integrity (absence of fibrosis), and left ventricular dilation (≥10% increase in end-diastolic volume) (48, 49). Comparative studies using CMR have demonstrated that elite endurance athletes exhibit greater LVWT, ventricular diameters, and volumes than non-athletes (50). Gender differences have also been observed, with male athletes generally showing larger cardiac mass and volume than females, while age and ethnicity further influence the degree of PCH (51). Importantly, CMR can detect subtle myocardial alterations, including fibrosis and edema, which are essential for distinguishing PCH from PMH. Despite these advantages, limitations such as high cost, long examination times, and the requirement for patient cooperation restrict its application in large-scale population screening (52).

#### 2.2.2 Laboratory evaluation

Laboratory assessment of PCH can be performed using myocardial biomarkers, which provide insights into cardiomyocyte function and remodeling. Commonly evaluated biomarkers include cardiac troponin (cTn), B-type natriuretic peptide (BNP), galectin-3 (Gal-3), and markers of ECM turnover. These indicators are valuable for detecting subtle changes in myocardial stress, injury, and remodeling, thereby complementing imaging techniques in the evaluation of PCH.

cTn comprises three subunits: troponin T (cTnT), troponin I (cTnI), and troponin C (cTnC). Of these, cTnT and cTnI exhibit high specificity and sensitivity for detecting myocardial injury or functional alterations. Under resting physiological conditions, circulating levels of cTnT and cTnI are minimal. However, during physical exertion, increased oxidative stress and changes in myocardial membrane permeability may facilitate the transient release of these proteins into the bloodstream. This phenomenon is attributed to reversible cellular stress rather than irreversible cardiomyocyte damage (53–55). Reported reference values for cTnT and cTnI in healthy individuals typically range from 0.001 to 0.05 ng/ml, although variations can occur depending on the analytical method, age, and sex (56).

For instance, a study examining cTnT concentrations in cyclists following varying exercise intensities demonstrated that moderate and high-intensity exertion led to a significant elevation in cTnT levels, whereas low-intensity activity produced no notable change. Importantly, cTnT levels returned to baseline after a recovery period, suggesting that such elevations reflect physiological responses to exercise rather than pathological myocardial injury (53). During moderate-to-high-intensity exercise, sustained mechanical stress on the cellular phospholipid bilayer results in localized membrane injury. The synthesis of membrane repair protein driven by IGF-1/PI3K/Akt pathway cannot fully keep pace with the rate of damage, leading to transient release of cTn. Concurrently, heightened energy demand excessively activates fatty acid oxidation (FAO) pathways, promoting the influx of free fatty acids (FFAs) into mitochondria and triggering excessive production of reactive oxygen species (ROS). ROS oxidize phospholipids such as phosphatidylcholine, destabilizing membrane integrity and facilitating the efflux of cTnT and cTnI through disrupted membrane gaps, ultimately leading to marked elevation of their serum concentrations (57, 58). It is noteworthy that cTn levels typically peak within 1–4 h following exercise and gradually return to baseline within 24–72 h. The magnitude of elevation and recovery kinetics, however, vary according to exercise modality. Endurance training is often associated with a pronounced rise in cTn and a prolonged normalization period, whereas resistance training generally induces a more modest elevation with a faster return to baseline (59, 60).

BNP is an endogenous hormone predominantly secreted by ventricular cardiomyocytes in response to increased wall tension. It exerts natriuretic, diuretic, and vasodilatory effects, with its dynamic changes regulated by the concurrent modulation of the CaN/NFAT pathway and FAO metabolic pathways activated by exercise. These changes reflect the cardiac adaptation to exercise (29, 61). In healthy individuals at rest, BNP levels are generally below 100 pg/ml. During exercise-induced PCH, increased ventricular wall tension leads to elevated intracellular Ca^2+^ concentrations, which bind to calmodulin (CaM) and activate CaN. CaN catalyzes the dephosphorylation of NFAT, which translocated to the nucleus and forms a complex with GATA binding protein 4 (GATA4), significantly enhancing BNP gene expression. Additionally, adrenergic signaling, mitogen-activated protein kinase (MAPK) pathways, and FAO-related signaling also contribute to this regulatory process. Under these signals, BNP secretion increases. This transient elevation, typically remaining below 200 pg/ml, is considered a normal physiological response and is not associated with adverse clinical outcomes in most cases. However, sustained BNP levels ≥300 pg/ml, especially when accompanied by clinical symptoms, may warrant further evaluation for potential cardiac dysfunction (62, 63). For example, Hosseini et al. reported elevated BNP concentrations in soccer players following competitive matches, although the levels remained well below thresholds indicative of heart failure (64). Interestingly, longitudinal observations have shown that serum BNP concentrations tend to decrease with increased cumulative training duration, suggesting an adaptive remodeling process in cardiac function in response to prolonged athletic activity (65). Furthermore, BNP typically normalizes more rapidly than cTn after exercise. Evidence indicates that BNP levels peak within 1–4 h post-exercise and return to baseline within 24 h. Notably, endurance exercise induces a greater elevation in BNP compared with resistance training, largely due to its volume overload stimulus. Despite differences in recovery kinetics between exercise modalities, these responses remain within the spectrum of physiological regulation (66, 67).

Gal-3 is both a fibrotic mediator and an inflammatory regulator, playing a pivotal role in diverse physiological and pathological processes (68). In healthy adults, normal serum Gal-3 levels typically range from 11.8 to 16.3 ng/ml, though values vary with detection method, age, and sex (69, 70). In older adults, downregulation of miR-222 reduces its inhibitory effect on Homeobox Containing 1 (Hmbox1), leading to the activation of the Transforming Growth Factor-β (TGF-β)/Smad pathway and the accumulation of inflammation and fibrosis, resulting in slight elevation of serum Gal-3 (71, 72). Research has shown that during exercise-induced PCH, the IGF-1/PI3K/Akt pathway activation results in phosphorylation of specific serine/threonine residues on Fork head Box O (FOXO), which helps suppress pro-inflammatory cytokine release, thereby reducing macrophage recruitment and activation and subsequently lowering Gal-3 production. Moreover, upregulation of miR-222 during exercise inhibits Hmbox1 mRNA expression, further suppressing TGF-β/Smad pathway activation and reducing Gal-3 production in fibroblasts. In PCH, serum Gal-3 levels remain normal or slightly reduced. In contrast, PMH, with insufficient IGF-1/PI3K/Akt pathway activation, leads to substantial secretion of Gal-3 from macrophages and fibroblasts, resulting in significantly elevated serum levels (73–76).

ECM turnover markers are biological indicators reflecting the dynamic balance between ECM synthesis and degradation. These markers are critical for assessing tissue fibrosis, inflammatory status, and related pathological conditions (77). In the context of PCH, ECM turnover markers aid in distinguishing physiological cardiac adaptation from pathological fibrosis. Commonly measured biomarkers include Procollagen Type I N-Terminal Propertied (PINP), Procollagen Type III N-Terminal Propertied (PIIINP), and Matrix Metalloproteinase-9 (MMP-9) (78). For reference, serum PINP and PIIINP levels in healthy adults are approximately 22–75 ng/ml (electrochemiluminescence immunoassay) and 2.5–5.5 ng/ml (radioimmunoassay), respectively, though values may vary with age, exercise, and assay methodology (79, 80). In PCH, PINP and PIIINP levels typically remain within the normal range or exhibit only mild, transient increases following exercise (81). Slight increases in PINP and PIIINP levels are likely due to the activation of signaling pathways such as Hippo-YAP, IGF-1/PI3K/Akt, and RNA m^6^A methylation, promoting protein synthesis and cellular growth. These pathways may also prevent pathological fibrosis by blocking TGF-β/Smad signaling and downregulating ANP/BNP expression. As a result, fibroblasts maintain a moderate activation state, synthesizing type I and III collagen, which ultimately leads to a mild elevation in serum PINP and PIIINP levels (81–83).

These elevations generally peak within hours post-exercise and return to baseline within 24–72 h, indicating that ECM remodeling during PCH is reversible and represents a normal physiological adaptation. By contrast, in PMH, PINP and PIIINP levels remain persistently elevated, reflecting sustained fibrosis and impaired diastolic function (84, 85). Notably, the response of ECM turnover markers to exercise differs from that of cTn and BNP, as ECM markers show a more moderate increase with a longer recovery period, suggesting that ECM remodeling is a sustained, rather than acute, process. These dynamic changes provide a valuable molecular-level tool for differentiating PCH from PMH.

#### 2.2.3 Electrophysiological assessment

The electrocardiogram (ECG) is a non-invasive diagnostic method that captures the heart's electrical activity via surface electrodes, translating the depolarization and repolarization of myocardial cells into characteristic waveform patterns. It serves as a crucial tool for assessing physiological and pathological changes in PCH (86, 87).

During exercise-induced PCH, several characteristic ECG alterations can be observed, among which increased QRS complex amplitude is particularly common (88). In the precordial leads, voltage augmentation may be evident in RV5 or RV6, exceeding the normal upper limit of 2.5 mV. In the limb leads, the R wave amplitude in lead aVL can surpass 12 mm, the R or S wave in lead I may exceed 15 mm, and the R wave in lead aVF may also be greater than 15 mm. A distinctive pattern is when the voltage in RV6 exceeds that in RV5 (S-type), which is attributed to an enhanced myocardial depolarization vector associated with PCH (89–91). Studies analyzing ECG data from endurance athletes with prolonged training have shown that increased QRS amplitude is common and significantly correlates with both the duration and intensity of training (92). ST-T segment abnormalities constitute another frequent ECG feature of PCH. In leads predominantly displaying R waves, T waves may appear deeply inverted, biphasic, or upright, while the ST segment can demonstrate horizontal or upward sloping elevation (typically >0.1 mV) or downward sloping depression. Marked elevation of V3 and V4, accompanied by upward sloping ST segments and upright T waves, reflects altered myocardial repolarization induced by PCH (93, 94). Observations in elite athletes following high-intensity training indicate that ST segment elevation with increased T wave amplitude in leads V3–V5 is common, and these training-induced changes typically normalize after a period of rest (95).

It is important to recognize the limitations of ECG in the assessment of PCH. Firstly, the ECG alterations described above are not specific to PCH; similar increases in QRS complex amplitude can also occur in conditions such as hypertensive heart disease and hypertrophic cardiomyopathy, necessitating careful differential diagnosis (96, 97). Secondly, in mild cases of PCH, the ECG may appear normal, potentially resulting in missed diagnoses. Moreover, although ECG reflects myocardial electrical conduction, it does not directly quantify left ventricular wall thickness, and its accuracy can be affected by factors such as heart rate, body size, and comorbid cardiac conditions (98). Consequently, the evaluation of PCH should be comprehensive, integrating medical history, clinical symptoms, physical findings, and complementary diagnostic tests (Table 2).

**Table 2:** Evaluation indicators of PCH.

Category	Evaluation technique/method	Parameter	Normal range	Changes observed in PCH	Relevant findings/remarks	References
Radiologic evaluation	3DE	LVEF	55%–70%	Normal or slightly elevated	LV cavity dilation leads to increased LVEDV	(13, 34, 35)
		LVM	Male: 73–115 g; Female: 62–95 g	Slightly above normal range	Considerable individual variation; does not reach PMH criteria	(35, 37, 38)
		LVMI	Male: ≤115 g/m^2^; Female: ≤95 g/m^2^	Slightly above normal range	Considerable individual variation; does not reach PMH criteria	(35, 37, 38)
	TDI	s’ wave	7–10 cm/s	Normal or slightly increased	Significantly correlated with LVEF	(40, 43)
		E/e’ ratio	<8 (normal); 8–15 (borderline)	Slightly elevated due to compensatory mechanisms	Possibly associated with enhanced myocardial contractility	(44–46)
	CMR	LVWT,LVST	LVWT: 9–10 mm, LVST: 10–12 mm	Mild increase	May be related to increased hemodynamics and metabolic adaptations	(48–50)
Laboratory evaluation	Myocardial biomarkers	cTnT, cTnI	0.001–0.05 ng/ml	Temporarily elevated after moderate to high-intensity exercise	Increased membrane permeability of myocardial cells	(53–56, 99)
		BNP	<100 pg/ml	Elevated post-exercise (generally ≤200 pg/ml)	Elevated ventricular wall stress	(62)
		Gal-3	11.8–16.3 ng/ml	Normal or mildly reduced	Reduced macrophage recruitment and activation, enhanced antioxidant capacity	(69, 70, 74, 75)
		PINP、PIIINP	PINP: 22–75 μg/L; PIIINP: 2.5–5.5 ng/ml	Normal or mildly increased	Rapid recovery after rest and adjustment	(79, 80, 84, 85)
Electrophysiological assessment	ECG	Amplitude of the QRS complex	RV5/RV6 ≤2.5 mV; R wave in lead aVL ≤12 mm; R/S ratio in lead I ≤15 mm; R wave in lead AVF ≤15 mm	RV5/RV6 >2.5 mV; R wave in lead aVL >12 mm; R/S ratio in lead I >15 mm; R wave in lead AVF >15 mm	This may be associated with an increase in myocardial depolarization vector, and is also related to the intensity and duration of training	(88–91)
		ST-T segment changes	No elevation of the ST segment; upright T wave	ST segment: upward sloping elevation or downward sloping depression; T wave: deeply inverted, biphasic, or upright	Associated with abnormalities in the myocardial repolarization process, which may be alleviated with rest and adjustment	(93, 94)

## 3 The relationship between exercise modalities and PCH

### 3.1 Single exercise modality-induced PCH

#### 3.1.1 Endurance exercise

Endurance exercise is characterized by sustained, moderate-intensity physical activity performed over an extended duration, requiring the integrated function of the cardiovascular and skeletal muscle systems (100). During prolonged endurance activities such as running, cycling, or swimming, the primary hemodynamic adaptations include increased heart rate and SV, resulting in a marked elevation of cardiac output. Simultaneously, enhanced skeletal muscle contraction and respiratory effort augment venous return to the heart. In response to this chronic volume overload, the heart undergoes structural remodeling, manifested primarily as chamber dilation and the progressive development of eccentric hypertrophy (101, 102). Parry-Williams et al. reported that athletes engaged in long-term endurance training exhibit cardiac changes including thickening of the left ventricular wall and chamber enlargement, with these adaptations being more prominent in older athletes (103). Similarly, research by the American Physiological Society demonstrated that one year of continuous endurance training resulted in increased the LVEDV and the LVM, along with enhanced myocardial contractility, while ventricular wall thickness remained largely unchanged. These findings suggest that endurance training promotes eccentric hypertrophic remodeling rather than concentric thickening of the myocardium (104). Experimental studies in animal models support these findings; for instance, rats subjected to daily swimming (60 min per session, five times per week) for 10 weeks developed eccentric cardiac hypertrophy, with the degree of remodeling positively correlated with exercise frequency (105). Mechanistically, the sustained elevation in venous return imposes chronic diastolic wall tension, triggering elongation of sarcomeres—the fundamental contractile units of cardiomyocytes—along the longitudinal axis. This leads to an increase in cardiomyocyte length without a corresponding increase in cell diameter, characteristic of eccentric hypertrophy (106–108).

Although long-term endurance exercise confers numerous cardiovascular benefits, the relationship between exercise intensity, duration, and the threshold for maladaptive cardiac remodeling remains controversial. Increasing evidence indicates that prolonged excessive endurance training may exert detrimental effects on cardiac structure and function. In a study of 40 athletes participating in high-intensity endurance events, such as marathons and triathlons, post-race assessments revealed a marked reduction in right ventricular ejection fraction (RVEF) alongside elevated circulating cardiac troponin (cTn) levels, with a linear correlation between the two. Further myocardial evaluation showed that 12% of these athletes exhibited myocardial fibrosis, and this subgroup had accumulated more years of endurance training compared to their counterparts (109). These findings underscore that excessive endurance exercise can negatively impact cardiac health, particularly affecting the right ventricle. A study in rats reported that 16 weeks of high-intensity training induced eccentric hypertrophy, accompanied by left ventricular diastolic dysfunction and significant myocardial fibrosis in the atria and right ventricle. However, these changes were reversible after the cessation of exercise, suggesting that an excessive volume load may surpass the heart's adaptive capacity, potentially triggering a transition from physiological remodeling to pathological cardiac changes (107). Moreover, when exercise load consistently exceeds the physiological tolerance of the body, it leads to sustained elevation of mTORC1 activity, resulting in endoplasmic reticulum (ER) homeostasis imbalance. This condition also triggers a reduction in miR-222 expression, thereby weakening its inhibitory effect on downstream *Cyclin-dependent kinase inhibitor 1B (p27)* and Hmbox1. This shift signifies the transition of the heart from adaptive remodeling to pathological remodeling, laying the molecular foundation for myocardial fibrosis and electrical activity abnormalities (110, 111). Therefore, quantifying these molecular biomarkers and dynamically adjusting exercise protocols based on the results ensures that cardiac remodeling remains within physiological adaptation limits, helping to prevent adverse cardiac remodeling due to overtraining. This approach forms the foundation for scientific management of athlete cardiac health.

#### 3.1.2 Resistance exercise

Resistance exercise involves active muscle contractions against external loads, aiming to enhance muscular strength, endurance, or hypertrophy. This form of exercise exerts beneficial effects not only on skeletal muscle but also on myocardial structure and function (112). During resistance training, sustained muscular contraction—often accompanied by relative hypoxia—leads to a marked increase in peripheral vascular resistance. This results in acute elevation in blood pressure and significantly augments left ventricular afterload. Over time, these hemodynamic changes stimulate myocardial remodeling, characterized by increased ventricular wall thickness without substantial chamber dilation—a process defined as concentric hypertrophy (113). Research has shown that after a 24-week resistance training program, consisting of three one-hour sessions per week, participants showed an increase in left ventricular mass (LVM) accompanied by ventricular wall thickening compared to baseline. These adaptations resulted in the development of concentric cardiac hypertrophy (114). In an animal study, 12 weeks of resistance training led to progressive increases in LVM, with observed increments of 8%, 12%, and 16% in weeks 4, 8, and 12, respectively. Importantly, these structural adaptations were accompanied by improved left ventricular systolic function, whereas changes in the LVEDV remained minimal. These findings support the development of concentric hypertrophy, with morphological changes becoming more pronounced over time (115). It is noteworthy that, while substantial evidence supports the induction of concentric hypertrophy by long-term resistance training, some human studies indicate that structural myocardial changes may be minimal even under significant afterload. For instance, a study involving participants with a mean age of approximately 68 years reported no significant alterations in ventricular size or wall thickness after 16 weeks of resistance training compared with baseline measurements. This observation may be attributed to age-related decline in cardiac autonomic regulation, which could diminish the myocardium's responsiveness to sympathetic stimulation (51, 114). Mechanistically, the primary driver of ventricular wall thickening in resistance exercise-induced concentric hypertrophy is the parallel addition of sarcomeres within cardiomyocytes. This pattern contrasts with the longitudinal sarcomere elongation seen in eccentric hypertrophy associated with endurance exercise (115).

**Figure 1:**
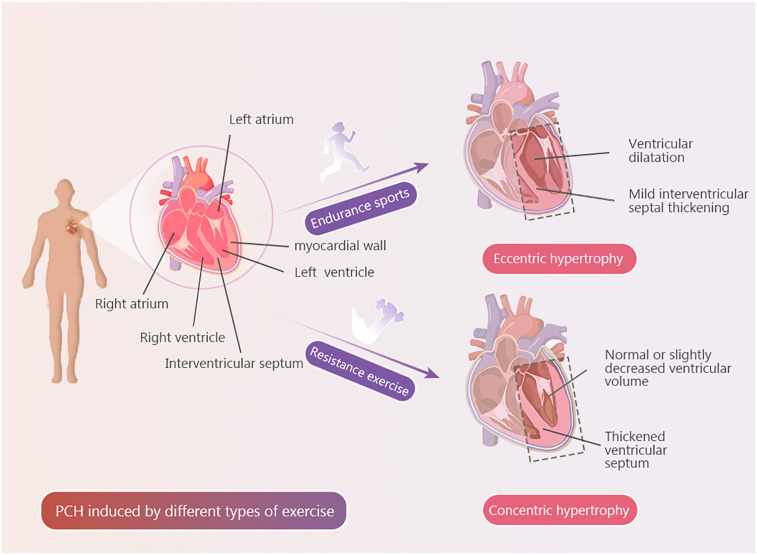
Exercise types and PCH. Explanatory text PCH induced by a single type of exercise.

During resistance exercise, sustained pressure overload induces adaptive cardiovascular remodeling, often manifested as transient yet substantial elevations in arterial blood pressure. Studies have shown that during maximal-intensity training, such as heavy weightlifting, arterial pressures can rise markedly compared to resting values (116). While this form of pressure overload is physiological, it bears resemblance to pathological pressure overload seen in conditions such as hypertension, which can also lead to concentric hypertrophy. However, pathologically induced hypertrophy is typically accompanied by systolic and/or diastolic dysfunction, as well as asymmetric thickening of the interventricular septum and the left ventricular posterior wall (117). The morphological similarities between physiological concentric hypertrophy resulting from resistance training and pathological forms of hypertrophy may pose diagnostic challenges and risk clinical misinterpretation (Figure 1).

### 3.2 Mixed exercise modality-induced PCH

Mixed exercise, which combines endurance and resistance training—such as high-intensity interval training (HIIT) and ball sports—provides alternating stimulation of distinct energy systems, thereby exerting a synergistic effect on the development of PCH. During the high-intensity resistance phases of HIIT, cardiac pressure load increases rapidly, promoting myocardial cell growth and hypertrophy. In contrast, the low-intensity endurance phases enhance blood circulation, ensuring sufficient delivery of oxygen and nutrients to myocardial cells. Additionally, metabolic by-products, such as lactate generated through anaerobic metabolism, are effectively cleared during these phases, supporting myocardial function and metabolic homeostasis (118, 119).

Given that most exercise or fitness programs incorporate both endurance and resistance training, exercise-induced PCH typically manifests as a combination of ventricular dilation and wall thickening, resulting in mixed hypertrophy. This represents an adaptive response of the heart to the imposed exercise load. Scharf et al. utilized magnetic resonance imaging (MRI) to evaluate the cardiac structure of elite triathletes, revealing significantly higher left ventricular end-diastolic volume (LVEDV) and left ventricular mass (LVM) compared with controls, while left atrial volume was inversely correlated with heart rate. The morphological and functional adaptations observed exhibited characteristics of both concentric and eccentric hypertrophy (120). Moreover, the relative proportion of endurance and resistance training within a mixed regimen differentially influences the pattern of PCH. Predominance of endurance exercise enhances myocardial aerobic capacity and cardiac endurance, improves diastolic function, and tends to produce an eccentric hypertrophy–like effect. In contrast, a greater emphasis on resistance exercise promotes myocardial contractility and increases ventricular wall thickness, favoring a concentric hypertrophy–like adaptation (121, 122). Supporting this, a longitudinal study of eight elite sailors (mean age 18) undergoing seven weeks of high-intensity HIIT demonstrated increased LVM and left ventricular wall thickness with minimal ventricular dilation, indicating the development of concentric hypertrophy. The extent of hypertrophy was significantly associated with total training volume and duration (123). Finally, the type and magnitude of exercise-induced PCH are modulated by individual factors, including age, sex, training history, and ethnicity. These findings highlight the importance of optimizing the ratio of endurance to resistance training in mixed exercise programs to maximize adaptive cardiac remodeling.

#### .3.3 Exercise intensity and PCH

Exercise intensity is commonly classified as low, moderate, or high, with each level exerting distinct physiological effects on the development of PCH. Low-intensity exercise is generally defined as physical activity that maintains heart rate at 50%–60% of an individual's maximum heart rate. This level of exertion is unlikely to induce significant cardiac remodeling and thus does not typically lead to the development of PCH. It is, however, considered appropriate for older adults or individuals with limited exercise tolerance and reduced physical capacity (124).

Moderate-intensity exercise is typically defined as activity that elevates heart rate to 60%–75% of an individual's maximum. This level of exertion increases venous return and cardiac preload, promoting volume overload and consequently inducing eccentric hypertrophy—characterized primarily by ventricular chamber dilation with minimal wall thickening. Moderate-intensity exercise is widely regarded as optimal for enhancing aerobic capacity and cardiorespiratory function and is considered the most effective stimulus for physiological eccentric hypertrophy (125, 126). In a study involving 12 participants (7 males and 5 females), a year-long moderate-intensity endurance training program was implemented. Cardiac magnetic imaging (CMI) was performed every three months to assess LVM, right ventricular mass (RVM), LVEDV, and right ventricular end-diastolic volume (RVEDV). The results demonstrated progressive increases in both cardiac mass and volume over the training period, indicating the development of eccentric hypertrophy (127). Experimental studies support this notion. After 8 weeks of moderate-intensity endurance training, rats in the exercise group exhibited significant increases in left and right ventricular chamber volumes and the LVM, with minimal changes in ventricular wall thickness—indicating the development of eccentric hypertrophy (128). Similarly, Fernandes et al. reported that rats subjected to 10 weeks of moderate-intensity swimming displayed load-induced eccentric remodeling of the myocardium (126). The underlying mechanism is believed to involve enhanced myocardial protein synthesis and suppressed protein degradation, leading to increased accumulation of myofibrils and cellular organelles within cardiomyocytes. These changes contribute to the enlargement of myocardial cells and the development of eccentric hypertrophy (129, 130). Additionally, moderate-intensity exercise promotes myocardial angiogenesis, improving coronary perfusion and facilitating the delivery of oxygen and nutrients to cardiomyocytes—thereby synergistically supporting cardiac growth and remodeling (131).

High-intensity exercise is defined as an activity that raises the heart rate above 75% of the individual's maximum, typically involving short-duration, high-energy expenditure efforts. This type of exercise exerts a dual effect on the heart, inducing both adaptive and potentially maladaptive changes (132). One prominent physiological adaptation is the development of concentric hypertrophy, marked by increased left ventricular wall thickness with minimal or no change in chamber volume (7). A study conducted by the Cellular Medicine Research Institute at Newcastle University examined the impact of HIIT on cardiac structure and function in patients with type 2 diabetes. After 12 weeks of HIIT or pharmacologic intervention, patients in the HIIT group exhibited greater left ventricular wall thickening, increased end-diastolic diameter, and improved myocardial contractility and relaxation compared to the pharmacologic treatment group, indicating the induction of concentric hypertrophy by HIIT (133). High-intensity exercise places substantial demands on cardiac output, stimulating robust protein synthesis in cardiomyocytes, which facilitates cellular growth and suppresses apoptosis (134). Furthermore, this training modality enhances myocardial energy metabolism, thereby contributing to the development of PCH (26). Nevertheless, prolonged or excessive acute high-intensity exercise may lead to electrical remodeling and right ventricular diastolic dysfunction, which could predispose individuals to atrial and ventricular arrhythmias (135) (Table 3).

**Table 3:** PCH induced by different exercise intensities.

Exercise intensity	Characteristics	Structural alteration	Physiological function changes	References
Low Intensity	Heart rate = 50–60% of maximum heart rate	No significant changes	Improved cardiovascular function, increased pumping capacity	(124, 136, 137)
Moderate Intensity	Heart rate = 60%–75% of maximum heart rate	Eccentric hypertrophy	Increased cardiac reserve, improved myocardial contractility	(125, 126, 129, 130)
High Intensity	Heart rate ≥75% of maximum heart rate	Concentric hypertrophy	Increased energy metabolism efficiency, significant improved pumping capacity in a short time	(7, 26, 132, 134)

## 4 Mechanisms of exercise-induced PCH

### 4.1 Transcriptional regulation

#### 4.1.1 The IGF-1/PI3K/Akt signaling pathway

During exercise-induced PCH, the IGF-1/ PI3K/Akt signaling pathway plays a central regulatory role. Activation of this pathway is associated with increased myofibril number, improved stroke volume (SV), and enhanced maximal oxygen uptake (VO_2_ max) (36). IGF-1, a polypeptide hormone structurally analogous to insulin, is primarily synthesized and secreted by the liver in response to growth hormone stimulation. It exerts multiple systemic effects, including the reduction of blood glucose and lipid levels (138). Studies have demonstrated that myocardial IGF-1 levels are elevated in athletes with PCH compared to those without hypertrophy, and serum IGF-1 concentrations increase following exercise, implicating IGF-1 as a key mediator in exercise-induced cardiac remodeling (139). During mechanical loading from exercise, mechanoreceptors in cardiomyocytes are activated, promoting IGF-1 binding to Insulin-like Growth Factor 1 Receptor (IGF-1R). This interaction triggers receptor autophosphorylation and subsequent recruitment of Insulin Receptor Substrate (IRS) proteins, which form a signaling complex that initiates PI3K activation (140).

The PI3K family comprises enzymes that phosphorylate phosphoinositide and are essential regulators of various cellular processes, including proliferation and apoptosis (141). In a study involving four weeks of swimming training in mice, those with reduced PI3K expression exhibited significantly attenuated PCH compared to controls, highlighting PI3K's critical role in exercise-induced cardiac remodeling (8). PI3K catalyzes the conversion of Phosphatidylinositol 4,5-bisphosphate (PIP_2_) to Phosphatidylinositol (3,4,5)-trisphosphate (PIP_3_) at the cell membrane. As a secondary messenger, PIP_3_ facilitates the membrane recruitment of Akt and Pyruvate Dehydrogenase Kinase 1 (PDK1) (142). Akt activation requires phosphorylation at T308 by PDK1 and at S473 by Mammalian target of rapamycin protein complex 2 (mTORC2), representing a pivotal step in the downstream signaling cascade. Evidence from an 8-week swimming protocol in rats showed a marked increase in phosphorylated Akt levels in myocardial tissue compared to sedentary controls. This activation of the PI3K/Akt/mTORC signaling axis was associated with the induction of PCH in response to exercise (143).

After Akt activation, it phosphorylates Tuberous Sclerosis Complex 2 *(TSC2)*, thereby inhibiting its suppressive effect on Ras Homolog Enriched in Brain-GTP (Rheb-GTP). This disinhibition allows Rheb-GTP to bind and activate the mTORC1 (144, 145). mTORC1 is a central serine/threonine kinase complex that phosphorylates key downstream effectors, including ribosomal protein S6 kinase 1 (S6K1) and eukaryotic translation initiation factor 4E-binding protein 1 (4E-BP1) (146). Phosphorylated S6K1 enhances ribosomal biogenesis and protein translation, thereby increasing intracellular protein synthesis, promoting cardiomyocyte hypertrophy, and facilitating the progression of PCH (147). Xu et al. further demonstrated that mTORC1 promotes protein biosynthesis primarily through 4E-BP1 phosphorylation (148). Once phosphorylated, 4E-BP1 detaches from eukaryotic translation initiation factor 4E (eIF4E), allowing eIF4E to interact with additional initiation factors to form the translation initiation complex. This process accelerates protein synthesis and leads to an accumulation of myofibrils and organelles within myocardial cells, laying the structural foundation for PCH development (144, 149).It is noteworthy that the activation level of the IGF-1/PI3K/Akt signaling pathway in response to exercise load is influenced by gender differences. Reports indicate that during exercise-induced PCH, female athletes typically exhibit lower activation levels of the IGF-1/PI3K/Akt pathway compared to their male counterparts, though the activation in females is more sustained (150, 151). This may be due to differences in myocardial cell responses to mechanical load and hormonal stimuli between genders.

#### 4.1.2 The NRG1/ErbB signaling pathway

Neuregulin 1 (NRG1) is an epidermal growth factor-like protein that promotes cardiomyocyte proliferation and hypertrophy while inhibiting apoptosis, playing a critical role in cardiac development and the maintenance of cardiac function (152, 153). Studies have shown that long-term aerobic exercise increases blood shear stress, which stimulates endothelial cells in the heart to secrete NRG1. The EGF-like domain of NRG1 binds to Erythroblastic Leukemia Viral Oncogene Homolog 4 (ErbB4) on the cell membrane, promoting the formation of ErbB4/ Erythroblastic Leukemia Viral Oncogene Homolog 2 (ErbB2) heterodimers (154). This process recruits downstream molecules such as Akt to the membrane and activates them. Through the PI3K/Akt/mTORC1 signaling pathway, it promotes the biosynthesis of proteins and ribosomes, facilitating myocardial cell hypertrophy and limited proliferation, thereby contributing to the development of PCH (155, 156). Additionally, studies have shown that, compared to younger athletes, older athletes exhibit significantly reduced expression of NRG1 and lower activity of the ErbB4 receptor in myocardial cells, which impacts hypertrophy and proliferation. This reduction is likely due to aging-related decline in myocardial cell responsiveness to load stimuli (157, 158).

Glycogen synthase kinase-3 beta (GSK-3β) is a multifunctional serine/threonine kinase involved in diverse cellular signaling pathways (159, 160). Following Akt activation, phosphorylation of GSK-3β at the Ser9 residue leads to suppression of its kinase activity, thereby attenuating the degradation of Beta-catenin (β-cat). The stabilized β-catenin accumulates in the cytoplasm and subsequently translocases into the nucleus, where it interacts with transcription factors such as T-cell factor (TCF) and lymphoid enhancer-binding factor (LEF). This complex activates transcription of downstream genes including Cellular Myelocytomatosis oncogene *(c-Myc)* and *Cyclin D1 (CCND1)*, thereby facilitating G1/S phase cell cycle progression and promoting cardiomyocyte growth and proliferation. This signaling cascade plays a pivotal role in exercise-induced PCH (161–163). Moreover, Akt has been shown to phosphorylate FOXO transcription factors at specific serine/threonine sites, promoting their association with 14-3-3 proteins and consequent nuclear export. This translocation suppresses the transcription of pro-apoptotic genes such as *Bcl-2 interacting cell death mediator (Bim)* and *Fas ligand (FasL)*, thereby inhibiting apoptosis and contributing to the preservation of cardiomyocyte viability. Together, these mechanisms support adaptive cardiac remodeling in response to exercise stimuli (73, 164).

#### 4.1.3 The Hippo-YAP signaling pathway

The biological output of the Hippo-YAP signaling pathway is primarily mediated through the transcriptional regulation of downstream target genes by Yes-associated protein (YAP) and transcriptional coactivator with PDZ-binding motif (TAZ). This pathway is critically involved in the development of exercise-induced PCH (165, 166). Under homeostatic conditions, the Hippo pathway remains constitutively active. Specifically, mammalian sterile 20-like kinases 1 and 2 (MST1/2) form a complex with Salvador homolog 1 (SAV1), which facilitates the phosphorylation and activation of large tumor suppressor kinases 1 and 2 (LATS1/2). Activated LATS1/2 subsequently phosphorylates YAP and TAZ, promoting their interaction with 14-3-3 proteins and sequestration in the cytoplasm. This cytoplasmic retention prevents YAP/TAZ from entering the nucleus and engaging in transcriptional activation of genes associated with cell proliferation and survival, thereby inhibiting cardiomyocyte growth and proliferation under non-stress conditions (167, 168).

In response to exercise-induced mechanical and biochemical cues, cardiomyocytes activate Rho-associated coiled-coil containing protein kinase (ROCK). Phosphorylated ROCK negatively regulates the Hippo pathway by inhibiting MST1/2 activity, thereby attenuating the activation of LATS1/2 kinases (169). Furthermore, exercise-induced mechanical stress, such as cardiomyocyte stretching, triggers cytoskeletal remodeling and the release of mechanosensitive signals that reduce LATS1/2 phosphorylation levels (170). As a result, the inactivation of LATS1/2 decreases phosphorylation at key regulatory sites on YAP (Ser127) and TAZ (Ser89), facilitating their nuclear translocation (171). Once translocated into the nucleus, YAP and TAZ interact with specific transcription factors to initiate the expression of target genes associated with cardiomyocyte growth and proliferation, including connective tissue growth factor (CTGF) and cysteine-rich angiogenic inducer 61 (CYR61) (172). These YAP/TAZ downstream effectors play distinct yet complementary roles in cardiac remodeling. CTGF contributes to creating a supportive extracellular environment for cardiomyocyte expansion, while CYR61 modulates cellular processes such as proliferation and survival, collectively promoting hypertrophic and proliferative responses in myocardial tissue and thereby facilitating the development of PCH (173, 174). Furthermore, exercise-induced activation of the IGF-1/PI3K/Akt signaling pathway has been reported to enable Akt to phosphorylate MST1/2 or inhibit LATS1/2 activity, resulting in decreased phosphorylation of YAP. This modulation initiates a cascade of downstream signaling events, which promotes myocardial cell growth and enhances cardiac contractility, thereby contributing to the development of PCH (84). It is important to note that with aging, the sensitivity of myocardial cells to mechanical stress gradually decreases, leading to diminished nuclear translocation of YAP. Consequently, older individuals often require higher-intensity load stimulation to induce PCH comparable to that of younger individuals (175).

#### 4.1.4 The C/EBP β-CITED4 signaling pathway

Exercise, through mechanical stress and metabolic regulation, activates intracellular signaling pathways such as IGF-1/PI3K/Akt and MAPK. These pathways subsequently activate downstream transcription factors, including cAMP response element binding protein (CREB). The cascade of downstream signaling molecules triggered by these transcription factors promotes protein synthesis within myocardial cells and increases myofibril number, playing a crucial role in the development of PCH (176). CREB has been reported to bind to the promoter region of the *CCAAT/enhancer-binding protein beta (C/EBPβ)* gene, leading to transcriptional repression of *C/EBPβ* mRNA expression (177). *C/EBPβ* is a transcription factor known to modulate genes involved in cell proliferation and differentiation (178). Experimental data indicate that aerobic exercise reduces *C/EBPβ* expression in cardiac tissue. For instance, after two weeks of aerobic training, mice exhibited significant downregulation of cardiac *C/EBPβ*. Moreover, cardiomyocyte-specific knockout of *C/EBPβ* in neonatal rat models promoted cardiomyocyte hypertrophy and proliferation (131). Comparable gene expression alterations have also been documented in zebrafish, mirroring the responses observed in murine models. In particular, Molkentin and colleagues demonstrated that swimming exercise suppressed *C/EBPβ* expression in zebrafish hearts, thereby enhancing cardiomyocyte growth and proliferation, promoting mild PCH, and increasing cardiomyocyte numbers following targeted *C/EBPβ* deletion (179). Similarly, endurance exercise-induced PCH in mice was associated with reduced *C/EBPβ* expression (180). Collectively, these findings support the notion that exercise-mediated downregulation of *C/EBPβ* serves as a critical mechanism contributing to cardiomyocyte proliferation and the adaptive remodeling characteristic of PCH.

Under resting conditions, *C/EBPβ* suppresses the transcriptional activity of the *CBP/p300-interacting trans activator with ED-rich tail 4 (CITED4)* gene by directly binding to its promoter region or by recruiting transcriptional repressors such as histone deacetylases (HDACs) (13, 181). *CITED4* functions as a transcriptional co-activator, interacting with co-activators like CBP/p300 to enhance the expression of downstream target genes (182). Exercise-induced downregulation of *C/EBPβ* expression has been shown to alleviate this repression, thereby facilitating the upregulation of *CITED4*. The increased expression of *CITED4* subsequently promotes cardiomyocyte proliferation and hypertrophy, inhibits apoptotic signaling pathways, and contributes to the development of PCH (107, 180). Furthermore, the regulation of the IGF-1/PI3K/Akt pathway has been shown to exhibit racial differences. Compared to European populations, East Asian populations have a lower activation threshold of CITED4 under exercise load due to genetic factors, requiring only moderate-intensity exercise to induce cardiac chamber enlargement. In contrast, African populations require higher-intensity exercise to effectively downregulate C/EBP β expression and produce significant regulatory effects (183–185). These differences highlight the need for personalized exercise protocols that consider genetic background to optimize cardiac adaptive remodeling.

#### 4.1.5 The CaN/NFAT signaling pathway

Mechanical stress imposed on cardiomyocytes during exercise leads to membrane depolarization, which activates voltage-gated L-type calcium channel (VGCC). This facilitates the influx of extracellular Ca^2+^, subsequently triggering ryanodine receptor (RyR) activation on the sarcoplasmic reticulum and promoting a substantial release of stored Ca^2+^ into the cytoplasm. As a result, intracellular Ca^2+^ concentrations rise markedly (186, 187). This calcium surge serves as a central initiator of the calcineurin–nuclear factor of activated T-cells (CaN/*NFAT*) signaling pathway (188). Ca^2+^ binds to CaM, forming a Ca^2+^/CaM complex that activates CaN, a calcium/calmodulin-dependent phosphatase. Activated CaN dephosphorylates serine residues on *NFAT* proteins, inducing a conformational change that reveals the nuclear localization signal (NLS). This modification enables *NFAT* translocation into the nucleus, where it regulates the transcription of target genes involved in cardiac remodeling and hypertrophy (189, 190).

Evidence indicates that *NFAT,* in cooperation with the transcription factor GATA4, binds to the promoter region of the *β-myosin heavy chain (β-MHC)* gene to enhance its transcriptional activity. This interaction facilitates an increase in cardiomyocyte volume while preserving contractile function, thereby contributing to adaptive cardiac remodeling without inducing fibrosis—a hallmark of exercise-induced PCH (191, 192). Moreover, atrial natriuretic peptide (ANP) and BNP are cardiac-derived peptide hormones with established roles in promoting natriuresis, diuresis, and vasodilation, thereby maintaining cardiovascular and fluid homeostasis (61, 193). Interestingly, studies have shown that nuclear-localized *NFAT* can bind to the promoter regions of the ANP and BNP genes and suppress their transcription. This inhibitory regulation is thought to help preserve myocardial metabolic balance and structural integrity, reducing the risk of ECM accumulation, fibrosis, and pathological remodeling under conditions of physiological stress such as exercise (194).

### 4.2 Post-transcriptional regulation

#### 4.2.1 RNA m^6^A methylation modification

During exercise-induced PCH, RNA N6-methyladenosine (m6A) methylation serves as a key regulatory mechanism. This regulation involves methyltransferases, demethylases, and downstream signaling cascades, which collectively enhance protein synthesis in myocardial cells, increase myofibril number, and promote metabolic reprogramming. m^6^A methylation involves the addition of a methyl group to the nitrogen at the sixth position of adenosine residues within RNA transcripts, forming N6-methyladenosine. This epitranscriptomic modification plays a fundamental role in RNA metabolism, influencing processes such as RNA stability, splicing, translation, and degradation (195, 196).

During exercise-induced PCH, regulatory pathways such as mRNA degradation and transcriptional repression contribute to the downregulation of methyltransferase like 14 (METTL14) mRNA expression, resulting in decreased METTL14 synthesis (197). Moreover, exercise modulates METTL14 protein levels via mechanisms including translational repression and enhanced protein degradation, collectively leading to an overall reduction in m6A methylation. Notably, reduced m6A levels have been shown to facilitate the degradation of Pleckstrin homology domain leucine-rich repeat protein phosphatase 2 (PHLPP2) mRNA through the YTH domain family protein 2 (YTHDF2) reader, thereby suppressing its expression (198). The downregulation of Phlpp2 alleviates its inhibitory effect on Akt activity, consequently activating the IGF-1/PI3K/Akt signaling pathway. This activation promotes myocardial hypertrophy and proliferation while inhibiting apoptosis, culminating in coordinated ventricular wall thickening and cardiac dilation, which supports the development of PCH (197). Furthermore, previous studies have demonstrated that Akt can inhibit the activity of GSK-3β. This inhibition reduces the phosphorylation of *NFAT*, enabling *NFAT* to expose its nuclear localization signal and translocate into the nucleus. Nuclear *NFAT* binds to DNA and upregulates the expression of pro-survival genes, including *interleukin-2 (IL-2)* and *B-cell lymphoma-extra large (Bcl-xL)*, as well as factors related to myocardial proliferation such as *CITED4*. This cascade enhances myocardial protein synthesis and proliferation while suppressing apoptosis, playing a critical role in the physiological remodeling associated with PCH (188, 199).

In the context of exercise-induced PCH, RNA m^6^A methylation modification exhibits significant sex- and age-related differences in regulation. Studies have shown that, due to estrogen influence, female athletes have reduced METTL14 mRNA expression in myocardial cells at baseline, along with enhanced demethylase activity. As a result, baseline m6A methylation levels are lower in females compared to males. Through this regulatory mechanism, females are able to more effectively downregulate PHLPP2 expression after exercise, thus more efficiently activating the IGF-1/PI3K/Akt pathway to induce PCH (197)^.^ However, in older individuals, the downregulation of METTL14 mRNA during exercise is less pronounced, and compared to younger individuals, the overall reduction in m^6^A methylation is smaller. This limits the degradation efficiency of PHLPP2 mRNA, restricting Akt activation and downstream signal molecule expression (200). These differences in regulation suggest that females exhibit stronger metabolic adaptability, while older individuals require higher load thresholds to achieve equivalent cardiac remodeling effects as younger individuals. This finding provides molecular-level theoretical support for the development of personalized exercise programs.

#### 4.2.2 Non-coding RNA regulation

Circular Uridine (circUtrn), a circular RNA associated with cardioprotective effects, is significantly upregulated in response to exercise-induced stress. CircUtrn enhances the activity of protein phosphatase 5 (PP5) by directly interacting with its catalytic domain (201). PP5, a serine/threonine phosphatase, is involved in critical cellular processes such as DNA damage repair and cell cycle regulation (202). Recent studies have demonstrated that PP5 activates downstream mammalian target of mTORC1, thereby relieving its inhibitory effect on *CCND1*. This promotes cardiomyocyte cell cycle re-entry, supporting myocardial repair and regeneration (203). This mechanism closely parallels the adaptive mTORC1 activation observed during PCH, enhancing cardiac reserve while mitigating pathological fibrosis. In addition, circUtrn functions as a molecular sponge for microRNA-195-5p (miR-195-5p), a pro-apoptotic microRNA known to suppress anti-apoptotic genes such as *F-box and WD repeat domain-containing protein 7 (FBXW7)* and *Mitofusin 2 (MFN2)* (204). By sequestering miR-195-5p, circUtrn alleviates its repression of *FBXW7* and *MFN2*, thereby reducing cardiomyocyte apoptosis, promoting mitochondrial biogenesis, and enhancing energy metabolism. This multi-target regulatory mechanism aligns closely with the fundamental features of exercise-induced PCH, contributing to improved myocardial adaptability and function (205).

*p27* negatively regulates cell cycle progression by inhibiting the Cyclin-dependent kinase 2 (CDK2)/Cyclin E complex, thereby blocking the G1-to-S phase transition (206). Studies have shown that miR-222 can bind to the 3ʹ untranslated region (3ʹ UTR) of *p27* mRNA, suppressing its translation and leading to reduced *p27* protein levels. This alleviation of CDK2/Cyclin E inhibition facilitates G1/S transition, promotes cardiomyocyte proliferation, and contributes to the enhancement of cardiac function associated with PCH (207). In addition to its role in cell cycle regulation, miR-222 also targets Hmbox1, a transcription factor that activates the Transforming Growth Factor-*β* (TGF-*β*)/Smad signaling pathway. This pathway promotes fibroblast-to-myofibroblast conversion and excessive ECM deposition, culminating in myocardial fibrosis (208). By binding to the 3ʹ UTR of Hmbox1 mRNA, miR-222 downregulates Hmbox1 expression, thereby inhibiting Transforming Growth Factor-β (TGF-*β*)/Smad protein (Smad) pathway activation and preventing fibrotic remodeling of the myocardium (209). Moreover, miR-222 indirectly upregulates α-myosin heavy chain (α-MHC) expression while suppressing β-MHC expression through the downregulation of p27 and Hmbox1. This shift increases the α-MHC/β-MHC ratio, thereby enhancing myocardial contractility, inhibiting myocardial fibrosis, and counteracting pathological cardiac remodeling (110).

### 4.3 Metabolic regulation

#### 4.3.1 Fatty acid oxidation

FAO, a central pathway in myocardial energy metabolism, not only supplies energy during exercise but also modulates gene expression through its metabolic intermediates and associated signaling pathways, forming a complex regulatory network. This mechanism plays a critical role in the development of PCH, enhances exercise capacity, and delays the onset of fatigue. Upon exercise stimulation, epinephrine (EPI) binds to β-adrenergic receptors (βARs), activating adenylate cyclase (AC) and leading to the generation of cyclic adenosine monophosphate (cAMP). cAMP subsequently activates protein kinase A (PKA), a key effector in this cascade (210). Studies have shown that PKA can enhance the activity of the fatty acid transporter CD36, thereby promoting the transmembrane uptake of free fatty acids (FFAs) into cardiomyocytes (211). Once internalized, FFAs are converted into acyl-Coenzyme A (Acyl-CoA) by cytosolic acyl-CoA synthetase (ACS). Acyl-CoA is then transported into mitochondria via carnitine palmitoyl transferase 1 (CPT1), the rate-limiting enzyme of mitochondrial FAO. Within the mitochondria, Acyl-CoA undergoes β-oxidation to generate acetyl-Coenzyme A (Acetyl-CoA), as well as reduced cofactors NADH and FADH_2_. These products subsequently fuel the tricarboxylic acid (TCA) cycle and oxidative phosphorylation, supplying ATP to meet the high energy demands of cardiomyocytes (149, 150). Enhanced efficiency of this metabolic pathway is essential for supporting the increased energy requirements during PCH induced by exercise (212, 213). Additionally, during exercise-induced PCH, female athletes generally exhibit a higher degree of FAO metabolic dependency compared to male athletes. It has been reported that under the regulation of estrogen, activation of transcription factors such as PPARα in females enhances FAO capacity during exercise, which significantly distinguishes female myocardial cells from the glycolytic metabolism phenotype predominant in males (214). This metabolic difference aids in the enhanced mitochondrial biogenesis capacity of female athletes during long-term endurance exercise.

Additionally, accumulating evidence suggests that acetyl-Coenzyme A (Acetyl-CoA), a key metabolic intermediate and signaling molecule, plays an important role in the epigenetic regulation of gene expression during exercise-induced PCH. Elevated intracellular levels of Acetyl-CoA can activate histone acetyltransferases (HATs), such as p300, which catalyze the acetylation of histone H3 at lysine 9 (H3K9ac) and lysine 27 (H3K27ac). These histone modifications promote chromatin relaxation and transcriptional activation of growth-related genes, including *GATA4* and *Myocyte Enhancer Factor 2 (MEF2)*. Consequently, this process downregulates the expression of hypertrophy-related genes such as ANP and BNP, while inhibiting pro-fibrotic pathways like TGF-*β*. While promoting myocardial cell growth and proliferation, it also induces the development of PCH (215). Moreover, this epigenetic regulation supports cardiomyocyte growth and proliferation while simultaneously suppressing pro-fibrotic signaling pathways, including TGF-β, thereby facilitating the development of a physiological hypertrophic phenotype (197). Peroxisome proliferator-activated receptor alpha (PPARα), a nuclear transcription factor critical for FAO, is highly expressed in myocardial tissue. During exercise, Akt-mediated phosphorylation enhances the transcriptional activity of PPARα. Activated PPARα promotes the expression of key FAO enzymes such as CPT1 and medium-chain acyl-CoA dehydrogenase (MCAD), thereby accelerating mitochondrial FAO and supporting sustained ATP production in cardiomyocytes (216). This metabolic reprogramming shifts substrate preference toward fatty acids, improving energy efficiency while minimizing the accumulation of potentially toxic metabolic byproducts. Collectively, these molecular and metabolic adaptations contribute to the emergence of a PCH phenotype characterized by cardiomyocyte hypertrophy and proliferation, enhanced cardiac output, and resistance to pathological fibrosis.

#### 4.3.2 Glucose metabolism

During exercise-induced PCH, multiple molecular pathways and metabolic regulatory networks are involved in glucose metabolism, supporting the energy supply and adaptive growth of myocardial cells, as well as enhancing exercise capacity. Glucose transporter type 4 (GLUT4), localized in intracellular vesicles, is the principal glucose transporter in cardiomyocytes (217). Wende et al. demonstrated that GLUT4 plays a pivotal role in the cardiac metabolic adaptation to increased hemodynamic load during exercise (218). Notably, activation of Akt signaling during exercise leads to the phosphorylation of Akt substrate of 160kDa (AS160), a Rab GTPase-activating protein. Phosphorylated AS160 loses its GAP activity, allowing GLUT4-containing vesicles to translocate to the plasma membrane. This translocation markedly increases glucose uptake by cardiomyocytes, thereby boosting ATP production to meet the immediate energy demands of the heart under exercise-induced stress (219). Additionally, Akt has been shown to inhibit the activity of GSK-3β, thereby limiting its phosphorylation of glycogen synthase (GS), resulting in the dephosphorylation and activation of GS. This process promotes the conversion of glucose into glycogen, providing a metabolic foundation for myocardial cell proliferation and hypertrophy. It also plays a positive role in protein synthesis and the delay of fatigue onset (220, 221).

**Figure 2:**
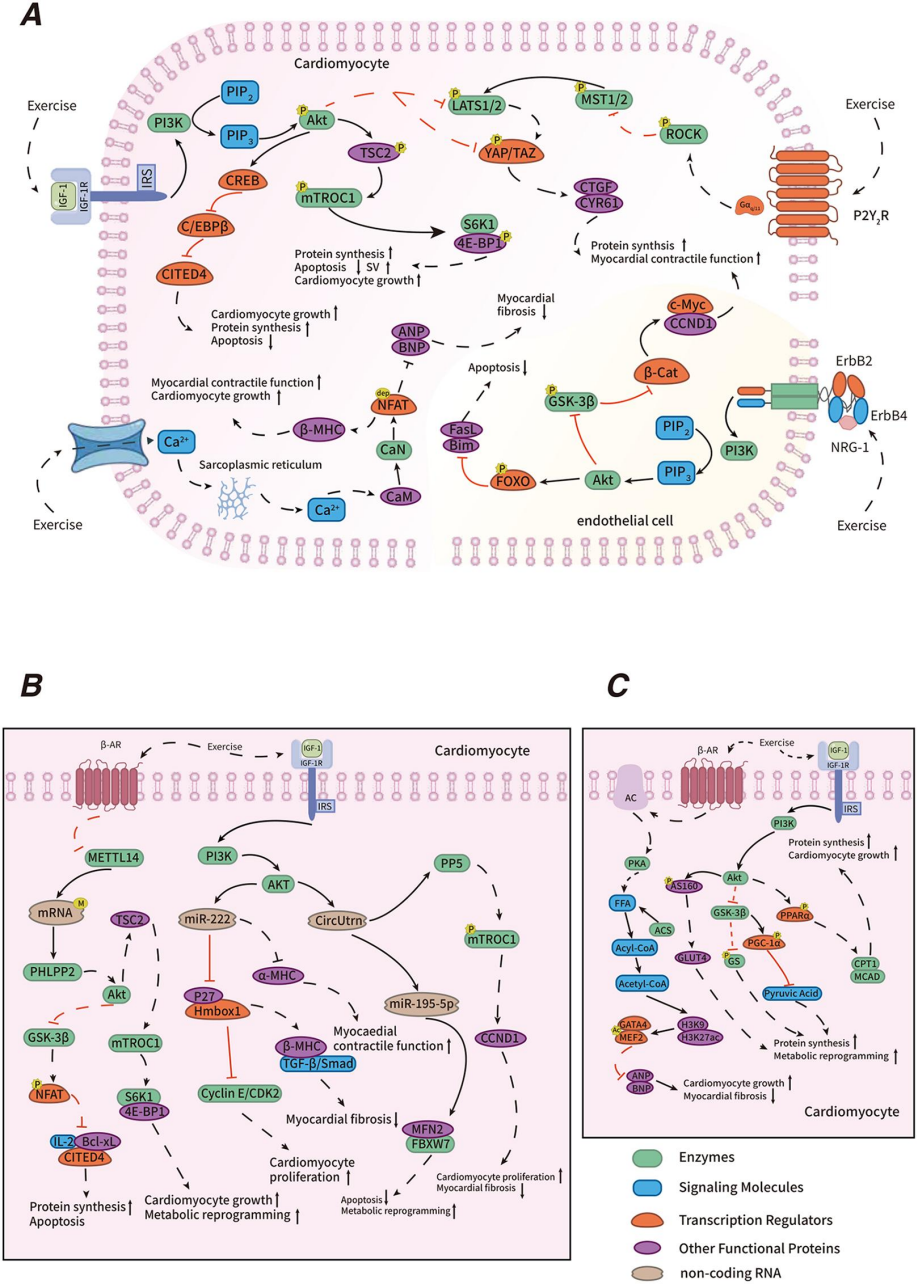
Mechanisms of exercise-induced PCH. Explanatory text: in the regulatory mechanism diagram of exercise-induced physiological cardiac hypertrophy, panel A illustrates transcriptional regulation, including the IGF-1/PI3K/Akt, NRG1/ErbB, Hippo-YAP, C/EBP β-CITED4, and CaN/NFAT signaling pathways. Panel B highlights post-transcriptional regulation, encompassing RNA m6A methylation modification and non-coding RNA regulation. Panel C depicts metabolic regulation, which includes fatty acid oxidation and glucose metabolism. Different colored entities in the diagram represent various functional biomolecules, where “P” denotes phosphorylation, “dep” denotes dephosphorylation, “M” denotes methylation, and “Ac” denotes acetylation. Solid lines indicate events that occur under normal physiological conditions, while dashed lines represent exercise-induced changes. Black lines indicate “promotion,” and red lines represent “suppression.” The symbols “
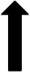
” and “
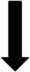
” indicate enhancement and attenuation, respectively.

Notably, Akt also promotes mitochondrial function by phosphorylating peroxisome proliferator-activated receptor gamma coactivator 1-alpha (PGC-1α), a master regulator of mitochondrial biogenesis. This phosphorylation event enhances the transcription of genes involved in mitochondrial DNA replication and oxidative metabolism, thereby improving mitochondrial efficiency and facilitating the complete oxidation of glycogen-derived substrates such as pyruvate. These processes provide sustained energy support, which is essential for the development of PCH (222, 223). In addition to mitochondrial adaptations, glucose metabolism is tightly regulated during PCH. Under normal physiological conditions, Hmbox1 acts as a negative regulator of glucose metabolism by inhibiting the expression of the *glucokinase gene (Gck)*. However, in a swimming-induced model of PCH, downregulation of Hmbox1 expression was observed during exercise, which led to the upregulation of *Gck*. Glucokinase catalyzes the phosphorylation of glucose to glucose-6-phosphate (G6P), the first and rate-limiting step in glycolysis. This enhances intracellular glucose uptake and utilization, ensuring a steady supply of energy substrates for cardiomyocyte metabolism. Such metabolic reprogramming is a hallmark of exercise-induced PCH and contributes to its adaptive, non-pathological nature (224) (Figure 2).

## 5 Positive effects of exercise-induced PCH on cardiovascular health

### 5.1 Improve cardiovascular health

Exercise, as a non-pharmacological intervention, supports cardiovascular health through diverse mechanisms, with PCH playing a pivotal role in this process (104). Exercise-induced PCH enhances cardiac function, a phenomenon closely linked to the remodeling of myocardial structure and the shift in MHC isoform expression. During aerobic exercise, a characteristic feature of PCH is an increased expression of α-MHC and a corresponding decrease in β-*MHC* expression in cardiomyocytes. Given that α-MHC possesses higher ATPase activity, this isoform switch contributes to improved contractile velocity and force generation (225). These adaptations lead to enhanced SV and a reduced resting heart rate, collectively improving circulatory efficiency and decreasing long-term cardiac workload. Additionally, studies have demonstrated that exercise-induced PCH results in increased cardiomyocyte volume and myofibril content, thereby enhancing both systolic and diastolic function. This structural remodeling enables the heart to more effectively meet the increased metabolic demands during physical activity by improving cardiac output and perfusion efficiency (115, 226). Moreover, PCH contributes to vascular benefits by improving endothelial function and reducing peripheral resistance, which supports the maintenance of stable blood pressure (107). Long-term exercise has been shown to stimulate nitric oxide (NO) release from endothelial cells, elevating intracellular cyclic guanosine monophosphate (cGMP) levels, which promotes vasodilation and decreases peripheral vascular resistance (227, 228). Moderate to high-intensity exercise, through the induction of PCH, is associated with reduced cardiovascular metabolic risk factors. These include lowered blood pressure and blood glucose levels, as well as decreased abdominal fat accumulation—factors that collectively aid in the prevention of cardiovascular diseases (107, 133).

### 5.2 Treatment of CVDs

Exercise-induced PCH also exerts beneficial effects in the prevention and treatment of CVD. Relevant studies have demonstrated that, after the random allocation of patients with ischemic non-obstructive coronary artery disease into groups, those in the intervention group—who underwent an 8-week personalized exercise program based on cardiopulmonary exercise testing (CPET) data—showed a significant reduction in total myocardial ischemic burden compared to the control group. Furthermore, notable improvements in cardiopulmonary function were observed in the intervention group (14). A key mediator of this protective role is IGF-1, which is essential for cardiovascular health. Clinical studies have reported that elderly individuals with reduced serum IGF-1 levels are at increased risk for heart failure. Notably, exercise-induced PCH has been shown to elevate circulating IGF-1 levels, thereby reducing oxidative stress in cardiomyocytes and improving cardiac function following myocardial infarction (229). In addition, circUtrn has emerged as a critical regulatory molecule in exercise-induced PCH, particularly in the context of myocardial ischemia-reperfusion (I/R) injury. In a mouse swimming training model, elevated circUtrn expression significantly reduced infarct size and enhanced cardiac function post-I/R injury. Conversely, suppression of circUtrn expression attenuated these protective effects, suggesting its potential as a biomarker and therapeutic target for coronary artery disease (201). Exercise-induced PCH also contributes to the modulation of cardiac electrophysiology. During PCH development, the myocardial action potential refractory period undergoes adaptive changes. It has been reported that exercise extends the effective refractory period, thereby reducing the susceptibility to ectopic excitations during this phase and lowering the risk of arrhythmia (230, 231). Furthermore, PCH promotes autonomic balance by modulating sympathetic and parasympathetic nervous system activity, which enhances cardiac rhythm stability and provides additional protection against arrhythmia (232, 233).

## 6 Challenges and future prospects

### 6.1 Molecular identification and clinical diagnosis challenges of PCH vs. PMH

Despite considerable advances in research on PCH, distinguishing it from PMH remains a major clinical and scientific challenge due to substantial overlaps in their macroscopic features and molecular profiles. Both PCH and PMH present with similar structural changes, including myocardial thickening and cardiac enlargement, and are often accompanied by comparable clinical symptoms such as palpitations, chest tightness, and shortness of breath. Consequently, differentiation based solely on gross morphology and clinical presentation is unreliable. At the molecular level, PCH and PMH share activation of key signaling pathways such as IGF-1/PI3K/Akt and MAPK, as well as similar alterations in the expression of cell cycle regulators, microRNAs, and other molecular markers. These parallels further complicate differentiation using standard molecular techniques (7, 229).

Current diagnostic modalities also have notable limitations. For instance, echocardiography, the most commonly used non-invasive imaging tool, can be affected by the heart's rhythmic motion, making it difficult to ensure consistent imaging planes and leading to measurement variability. Furthermore, echocardiography has limited spatial resolution for detecting subtle distinctions, such as differences in myocardial fiber orientation or the degree of interstitial fibrosis, which are critical for distinguishing PCH from PMH (234). Similarly, ECG offers limited diagnostic specificity. Both PCH and PMH can manifest as ST-T segment abnormalities, T-wave inversions, and signs of left ventricular hypertrophy, making it challenging to differentiate between them (235). In addition, confounding factors such as electrolyte imbalances and certain medications can induce ECG changes that mimic those seen in hypertrophy, further reducing diagnostic accuracy (236). TDI is primarily employed to evaluate myocardial diastolic function and assist in distinguishing types of MH. In the early stages of PCH, diastolic function at rest often remains normal, and early diastolic parameters in PMH may also appear unremarkable, complicating differentiation between the two. Moreover, TDI image acquisition and analysis require substantial expertise, and inter-operator variability can affect diagnostic accuracy (237). Strain Imaging (SI) also has limitations for early detection of PMH, particularly when ventricular wall thickening is minimal, as abnormal myocardial strain may not be evident. Physiological factors such as age, sex, and exercise level can further influence SI results, increasing the complexity of clinical interpretation. In addition, lack of standardization across different SI systems or manufacturers limits result comparability (238). Cardiac magnetic resonance (CMR) imaging likewise faces challenges in distinguishing MH subtypes. For example, transient metabolic enhancement may occur during early PCH, which can be difficult to differentiate from the metabolic abnormalities observed in early PMH. False-positive or false-negative findings may also arise, particularly in early or borderline cases, complicating accurate differentiation (239). Notably, following cessation of training, athletes typically exhibit gradual reductions in ventricular wall thickness, which can often be detected within a few months using three-dimensional echocardiography (3DE) and CMR. In contrast, in patients with PMH, ventricular wall thickness may remain unchanged or even progress despite exercise cessation, especially in the presence of other pathological factors (15, 240).

In clinical practice, especially when working with specialized populations such as elite athletes, there often exist ambiguous “gray zones” that are difficult to precisely define. For example, some elite athletes may have LVWT measurements in the borderline range of 13–15 mm, often accompanied by a slight increase in plasma BNP levels. This scenario can lead to diagnostic challenges (17). To address this, a novel multi-stage assessment system should be developed. For instance, if a 3DE reveals an LVWT of 13–15 mm, it should be categorized as a “gray zone.” This finding would necessitate the use of additional methods such as biomarkers, molecular markers, comprehensive assessments, and regular follow-up evaluations for multimodal assessment, thereby improving the accuracy of clinical evaluations (241, 242).

To address these challenges, future research should prioritize the development and refinement of diagnostic technologies with enhanced precision and specificity. The integration of artificial intelligence, machine learning, and big data analytics holds great promise for advancing diagnostic algorithms. These innovations could facilitate the creation of a more robust and accurate classification system for myocardial hypertrophy, ultimately supporting more effective and individualized clinical decision-making (Table 4).

**Table 4:** Comparative diagnosis of different testing techniques.

Technique	Sensitivity	Specificity	Pattern of Manifestation	References
3DE	Moderate-High	Moderate	PCH: Thickened myocardial walls, enlarged heart chamber; PMH: Non-thickened myocardial walls(e.9., significant increase in endocardial thickness)	(34, 243)
ECG	Low-Moderate	Low	PCH: Increase in QRS wave amplitude with no abnormalities; PMH: ST-T changes, QRS wave modification, etc., prominent abnormalities	(9, 92, 244)
TDI	High	Moderate-High	PCH: Normal or enhanced myocardial function.	(43, 245)
			PMH: Decreased myocardial function.	
SI	High	High	PCH: Significant residual increase.	(238, 246, 247)
			PMH: Decrease in response to changes, especially when compared to alterations.	
CMI	Extremely High	Extremely High	PCH: Increased serum albumin or increased egg white protein.	(48, 239)
			PMH: Changes in collagen synthesis or degradation.	

### 6.2 Overexercise-induced decompensation and its prevention

While exercise-induced PCH offers substantial cardiovascular benefits, its potential adverse effects should not be underestimated. Emerging evidence suggests that, in the early stages of PCH, the heart undergoes adaptive remodeling through compensatory mechanisms such as ventricular wall thickening and chamber dilation. During this phase, SV and ejection fraction may increase slightly. However, these compensatory adaptations are inherently limited. When they are no longer sufficient to meet the body's demands for oxygen and circulation, the heart may enter a decompensate state. This transition is marked by pathological remodeling, including an imbalance between collagen synthesis and degradation in the ECM, leading to myocardial fibrosis and impaired diastolic function. Without timely regulation or intervention, prolonged decompensation may ultimately lead to heart failure, posing a significant threat to health and survival (248). Additionally, cardiac decompensation disrupts the distribution and function of ion channels in cardiomyocytes, altering both the velocity and pattern of intercellular electrical conduction. These disturbances can impair myocardial excitability, automaticity, and conductivity, thereby increasing susceptibility to arrhythmias (249, 250).

Preventing the progression of cardiac decompensation is therefore of paramount importance. Effective strategies include the development of individualized exercise programs, pre-exercise assessments, real-time physiological monitoring during training, and structured recovery protocols (251, 252). In the development of exercise protocols, data from CPET should be systematically analyzed and incorporated into individualized exercise load assessments. Key indicators, such as VO_2_ max and anaerobic threshold, along with individual factors including age, health status, exercise capacity, and fitness goals, can be used to establish precise exercise intensity thresholds. Integration of genetic and metabolomic analyses may further inform individual responses to different exercise modalities, allowing for the precise tailoring of exercise programs. Exercise interventions should follow recommended frequencies and durations, with progressive increases in load to ensure safe adaptation (253, 254). Prior to implementation, a comprehensive evaluation of the individual's physical condition—including ECG, echocardiography, and other diagnostic assessments—provides a baseline understanding of cardiac function and overall health, forming the foundation for scientifically designed exercise protocols. During training, dynamic monitoring using wearable devices, which track lactate concentration measurement and continuous blood pressure, can provide real-time insights into autonomic nervous system function and cardiovascular load. This approach enables optimization of exercise intensity and mitigates the risk of cardiac decompensation (255, 256). Recent research has indicated that within wearable device-based health management systems, integrating physiological models with neural network methodologies for the development of personalized exercise programs can accurately predict the cardiovascular load responses of the body. This approach also allows for the quantification of environmental factors impacting heart health, such as elevated heart rate due to high-temperature and high-humidity environments. By providing real-time monitoring and assessment, this system can significantly contribute to preventing the onset of cardiac decompensation, thus enhancing overall cardiovascular health management (257). Following exercise, standardized cool-down protocols—including stretching and relaxation—are recommended to promote recovery and reduce fatigue (258). Moreover, lifestyle interventions such as optimizing nutritional intake and maintaining adequate sleep quality, in combination with pharmacological strategies, when necessary, can further mitigate the risk of decompensation (259).

Looking ahead, the implementation of personalized exercise prescriptions tailored to individual characteristics and needs will be essential for maximizing the cardiovascular benefits of physical activity while minimizing the risks. Concurrently, deeper investigation into the molecular mechanisms underlying cardiac decompensation will be critical for identifying novel therapeutic targets and developing targeted pharmacologic interventions, thereby advancing the precision prevention and management of exercise-induced cardiac injury.

## 7 Conclusion

Exercise-induced PCH represents an adaptive remodeling process reflecting the coordinated optimization of cardiac structure and function in response to regular exercise. This review systematically delineates the differential impacts of various exercise modalities on cardiac morphology and performance: endurance exercise predominantly induces eccentric hypertrophy via volume overload, whereas resistance exercise promotes concentric hypertrophy through pressure overload. The development of PCH is orchestrated by a complex interplay of regulatory mechanisms, including transcriptional, post-transcriptional, and metabolic pathways. Importantly, PCH exhibits both preventive and therapeutic potential for CVDs, such as hypertension and heart failure, by enhancing cardiac pump efficiency, optimizing energy metabolism, and mitigating myocardial fibrosis.

Despite these advances, several challenges remain. First, distinguishing PCH from PMH requires overcoming the limitations of current imaging modalities and biomarkers, necessitating the creation of precise, stratified diagnostic frameworks based on multimodal data integration. Second, the potential risk of cardiac decompensation induced by excessive exercise underscores the urgent need for a personalized, dynamic evaluation system for exercise intensity. Such a system should integrate genetic profiling, metabolic phenotyping, and real-time biosensor data to prevent adverse consequences like myocardial fibrosis and arrhythmias. Future research should prioritize elucidating the dose–response thresholds of exercise on cardiac adaptive remodeling and dissecting the molecular pathways that precipitate cardiac decompensation. These efforts may facilitate a paradigm shift from generalized “personalized exercise prescriptions” toward strategies aimed at “maximizing heart health,” thereby advancing cardiovascular wellness on a broader scale.

## Glossary

α-MHC: α-Myosin heavy chain
β-cat: β-catenin
β-MHC: β-Myosin heavy chain
βAR: β adrenergic receptor
3'UTR: 3' untranslated region
4E-BP1: Eukaryotic translation initiation factor 4E binding protein 1
AC: Adenyl cyclase
Acetyl-coA: Acetyl-Coenzyme A
ACS: Acyl-coA synthetase
Acyl-CoA: Acyl-coenzyme A
Akt: protein kinase B
ANP: atrial natriuretic peptide
AS160: Akt substrate of 160 kDa
Bcl-xL: B-cell iymphoma-extra large
Bim: B-cell lymphoma 2-interacting cell death mediator
BNP: brain natriuretic peptide
c-Myc: cellular myelocytomatosis oncogene
C/EBP β: CCAAT/enhancer binding protein beta
CaM: Calmodulin
cAMP: cyclic adenosine monophosphate
CaN: Calcineurin
CCND1: Cyclin D1
CDK2: cyclin-dependent kinase 2
circUtrn: circular uridine
CITED4: CBP/p300-interacting trans activator with ED-rich tail 4
CMR: cardiac magnetic resonance imaging
CPET: cardiopulmonary exercise testing.
CPT1: carnitine palmitoyltransferase 1
CREB: cAMP response element binding protein
CTGF: connective tissue growth factor
cTnC: Troponin C
cTnI: Troponin I
cTnT: Troponin T
CVD: cardiovascular diseases
CYR61: Cysteine-Rich angiogenic inducer 61
ECG: electrocardiogram
ECM: extracellular matrix
eIF4E: Eukaryotic translation initiation factor 4E
EPI: epinephrine
ErbB2: erythroblastic leukemia viral oncogene homolog 2
ErbB4: erythroblastic leukemia viral oncogene homolog 4
FAO: fatty acid oxidation
FasL: Fas Ligand
FBXW7: F-box and WD repeat domain-containing protein 7
FFA: free fatty acids
FOXO: forkhead box O
G6P: Glucose-6-Phosphate
Gal-3: Galectin-3
GATA4: GATA binding protein 4
Gck: glucokinase gene
GH: growth hormone
GLUT4: glucose transporter type 4
GS: glycogen synthase
GSK-3β: glycogen synthase kinase-3 beta
H3K27ac: acetylation of histone H3 at lysine 27
H3K9ac: acetylation of histone H3 at lysine 9
HATs: histone acetyltransferase
HDACs: histone deacetylases
HIIT: High-intensity Interval Training
Hmbox1: homeobox containing 1
IGF-1: insulin-like growth factor 1
IGF-1R: insulin-like growth factor 1 receptor
IL-2: Interleukin-2
IRS: insulin receptor substrate
LATS1/2: large tumor suppressor kinases 1 and 2
LEF: lymphoid enhancer factor
LVEDV: left ventricular end-diastolic volume
LVEF: left ventricular ejection fraction
LVFP: left ventricular filling pressure
LVM: left ventricular mass
LVMI: left ventricular mass index
LVST: interventricular septal thickness
LVWT: left ventricular wall thickness
m6A: N6-methyladenosine
MAPK: mitogen activated protein kinase
MCAD: medium-chain acyl-coA dehydrogenase
MEF2: myocyte enhancer factor 2
METTL14: Methyltransferase like 14
MFN2: Mitofusin 2
MH: myocardial hypertrophy
miR-195-5p: MicroRNA-195-5p
MMP-9: matrix metalloproteinase-9
MST1/2: mammalian sterile 20-like kinase 1/2
mTORC1: mammalian target of rapamycin protein complex 1
mTORC2: mammalian target of rapamycin protein complex 2
NFAT: nuclear factor of activated T-cells
NLS: nuclear localization signal
NRG1: Neuregulin 1
p27: cyclin-dependent kinase inhibitor 1B
PCH: physiological cardiac hypertrophy
PDK1: pyruvate dehydrogenase kinase 1
PGC-1α: peroxisome proliferator-activated receptor gamma coactivator 1-Alpha
PHLPP2: Pleckstrin homology domain leucine - rich repeat protein phosphatase 2
PI3K: Phosphatidylinositol 3-Kinase
PIIINP: procollagen type III N-Terminal propeptide
PINP: procollagen type I N-Terminal propeptide
PIP2: phosphatidylinositol 4,5-bisphosphate
PIP3: phosphatidylinositol (3,4,5)-trisphosphate
PKA: protein kinase A
PMH: pathological myocardial hypertrophy
PP5: protein phosphatase 5
PPARα: peroxisome proliferator-activated receptor alpha
Rheb GTP: Ras homolog enriched in brain guanosine triphosphate
ROCK: rho-associated coiled-coil containing protein kinase
RVEF: right ventricular ejection fraction
RyR: ryanodine receptor
S6K1: ribosomal protein S6 kinase 1
SAV1: Salvador homolog 1
SI: strain imaging
SV: stroke volume
TAZ: transcriptional coactivator with PDZ-binding motif
TCF: transcription factor 4
TF: transcription factor
TGF-β: transforming growth factor-β
TSC2: Tuberous Sclerosis Complex 2
VGCC: voltage-gated L-type calcium channel
YAP: yes-associated protein
YTHDF2: YTH domain family member 2
